# Consumer preference for dried mango attributes: A conjoint study among Dutch, Chinese, and Indonesian consumers

**DOI:** 10.1111/1750-3841.15439

**Published:** 2020-09-12

**Authors:** Ita Sulistyawati, Matthijs Dekker, Ruud Verkerk, Bea Steenbekkers

**Affiliations:** ^1^ Food Quality and Design Group Agrotechnology and Food Sciences Wageningen University & Research Bornse Weilanden 9 Wageningen 6708 WG the Netherlands; ^2^ Department of Food Technology Soegijapranata Catholic University Pawiyatan Luhur IV/1 Semarang 50234 Indonesia

**Keywords:** conjoint analysis, consumer, drying, fruit, product familiarity

## Abstract

**Abstract:**

One way to add value to tropical fruit and increase its availability in the global market is to develop new, less perishable, products from fresh fruit. The purpose of this study is to compare the perception of key quality attributes and preferences of dried mango between consumers with different familiarity and health consciousness. This study surveyed respondents from China, Indonesia, and the Netherlands via an adaptive choice‐based conjoint method (*n* = 483) to evaluate intrinsic quality attributes that influenced consumer preference for dried mango. Consumers in different countries have different texture, taste, and color preferences for dried mango. The most important attribute for the Dutch and Chinese was “free from extra ingredients”, while for Indonesians, it was the texture. Familiarity with dried mango and health consciousness do not influence consumer preference of intrinsic attributes of dried mango. Different preferences of intrinsic attributes of dried mango between countries are related to cultural differences. This study provides useful insights for food manufacturers into the significance of key intrinsic quality attributes in developing dried mango.

**Practical Application:**

Intrinsic quality parameters of dried mango are not perceived in the same way by every consumer and this is related to cultural differences. Crispy texture is important only for Indonesian consumers, while “free from extra ingredients” is the most important for Dutch and Chinese consumers. This information is relevant when developing dried mango products for the respective markets.

## 1 INTRODUCTION

The current demand for healthy and convenient food has increased, and consumers more often choose processed fruits, for example, dried fruits, jam, and fruit snacks (Grunert, 2013; Jesionkowska, Sijtsema, Simoneaux, Konopacka, & Plocharski, 2008; Sadler et al., 2019). To increase the value of fruits and its availability in the global market, it is necessary to develop products that meet consumer wishes and preferences of the intended markets (Grunert, 2005). This study contributes to the understanding of European and Asian consumer preferences for dried mango. The study assesses especially consumer preferences for the intrinsic quality of dried mango in relation to health (e.g., nutrition) and sensory.

The quality preference‐ specifically on health or sensory properties ‐ of some dried tropical fruits,for example, mango, pineapple, banana, kiwi, and litchi, has been investigated in European (Alphonce, Temu, & Almli, 2015; Bower & Ferguson, 2008) and Asian countries (Cinar, 2018; Precoppe et al., 2014). To our knowledge, no studies so far compared the quality preferences of dried tropical fruit between both regions. Asia is one of the predominant producers and suppliers of major traded tropical fruits,that is, mango, guava, and pineapple (FAO, 2019). Most of these fruits are destined for domestic markets, thus fresh tropical fruits are widely available. The European Union, meanwhile, is one of the largest import markets of fresh tropical fruits (FAO, 2019). The fruits are highly appreciated for their exotic appearance, health benefits, and tastefulness (Wismer, 2014; Yahia, García‐Solís, & Celis, 2019), yet most tropical fruits are seasonal. Processing fresh fruit into dried fruit is of global market interest since the dried product has longer availability and high versatility in various food products, including breakfast cereals, fruit bars, and mixed with nuts (CBI, 2019; Sadler et al., 2019). Previous consumer preference studies on dried fruits shown that health‐related attributes, such as nutritional content, a positive influence on health, and functional ingredients were among the important intrinsic attributes (Asioli et al., 2019; Jesionkowska, Sijtsema, Konopacka, & Symoneaux, 2009). Therefore, this study identified consumer preferences for health and sensory properties, especially at understanding which attributes might help to increase the value of dried mango for the intended markets. Both types of information are essential for consumer‐oriented product development to be successful (Grunert, 2005).

This study gives an account of the consumer's preference for food to be used in the early stages of product development, which is dominated by the intrinsic quality attributes of the product. The intrinsic attributes refer to those attributes “that cannot be changed or manipulated experimentally without at the same time modifying the physical characteristics of the product itself” (Olson & Jacoby, 1972). The preference is influenced by what they perceive from “experienced” intrinsic quality attributes (color, taste, flavor, and texture). This perception is used to assess other more “hidden” intrinsic quality attributes of the product, such as health (nutritional value and vitamins content), and to determine the overall quality of a food product (Asioli et al., 2017).

Among dried tropical fruits, dried mango poses an interesting case. Mango is one of the top three most consumed tropical fruits due to its attractive color, unique flavor, and its nutritional value (FAO, 2019). One way to increase the availability of and to add value to mango is by developing mango products, for example, dried mango. Product features like an extended shelf‐life, convenience, and “fresh‐like” characteristics are provided by dried fruit with maintained nutrients and health‐promoting value (Ciurzyńska, Kowalska, Czajkowska, & Lenart, 2016; Orsat, Changrue, & Vijaya Raghavan, 2006; Witrowa‐Rajchert, Wiktor, Sledz, & Nowacka, 2014).

Besides sensory and health properties of the product, product familiarity and health consciousness play a key role in consumers’ food choice, including fresh fruit (Pollard, Kirk, & Cade, 2002), processed tropical fruits (Sabbe, Verbeke, & Van Damme, 2008), and dried fruits (Almli, Asioli, & Rocha, 2019). Familiarity has been associated with personal product‐related experiences, such as knowledge, purchase, consumption, and product typicality,that is, to what extent the product represents its overall category (Bredahl, 2003; Frez‐Muñoz, Steenbekkers, & Fogliano, 2016; Park & Lessig, 1981). A higher familiarity with the product has been associated with a higher understanding of its attributes, which is translated to more informed product evaluations (Banović, Fontes, Barreira, & Grunert, 2012). Consumers with different familiarity levels assess both intrinsic and extrinsic attributes in different ways (Banović et al., 2012; Bredahl, 2003; Frez‐Muñoz et al., 2016).

Healthiness of the product is one of the main motives in consumers’ food choice (Grunert, 2013; Januszewska, Pieniak, & Verbeke, 2011; Milošević, Žeželj, Gorton, & Barjolle, 2012). Healthiness is often associated with sensory (flavor, taste, color, and texture) and natural content characteristics (no additives and natural ingredients) (Chambers, Chambers, & Castro, 2018; Puska & Luomala, 2016). According to the literature, health consciousness could influence how consumers assess the importance of attributes in a food product (Chen, 2013; Schifferstein & Ophuis Oude, 1998). This study aims to compare the perception of key quality attributes and their preferences for intrinsic characteristics of dried mango between consumers with different familiarity levels to support consumer‐oriented product development of dried mango. It is hypothesized that perceptions of key quality attributes are different among different familiarity levels. Second, it is hypothesized that the intrinsic quality attributes of dried mango are perceived differently by consumers with different levels of health consciousness. Therefore, a cross‐national online survey was conducted on preference of dried mango in three countries: China, Indonesia, and the Netherlands. A conjoint analysis was applied to identify the importance of different intrinsic quality attributes and the preference for the attribute levels of dried mango, as well as to evaluate if these attributes vary due to nationality, demographic data, and health consciousness of respondents.

## 2 MATERIALS AND METHODS

### 2.1 Conjoint analysis

#### 2.1.1 Selection of attributes and levels by focus groups

To determine the attributes and levels to be evaluated in the conjoint analysis, our previously obtained focus groups results were used by selecting the intrinsic attributes that were mentioned most often by participants as relevant to eat dried mango, Table 1 (Sulistyawati, Sijtsema, Dekker, Verkerk, & Steenbekkers, 2019). The most often‐mentioned intrinsic attributes were determined by applying content analysis to the translated verbatim transcription data using ATLAS.ti 7.5.12 for Windows. The focus groups showed that sweetening agents and (the absence of) extra ingredients were the most often mentioned intrinsic attributes related to perceived healthiness of dried mango, followed by taste, flavor, color, and texture. Similarly, the chosen levels for each of these attributes were based on the outcomes of the focus groups.

**Table 1:** Intrinsic attributes and their levels of dried mango used in the conjoint analysis

Extra ingredients	Sweetener	Texture
Salt	High calorie, sugar/honey	Chewy
Spices (e.g., chili and ginger)	Low calorie, natural sweetener	Soft
Combination of salt and spices	No calorie, artificial sweetener	Crispy
No extra ingredients		
**Color**	**Taste**	**Mango flavor**
Yellow	More sweet than sour	Weaker than fresh mango
Light orange	Balanced sweet and sour	Similar to fresh mango
Orange	More sour than sweet	Stronger than fresh mango
Intense orange		

#### 2.1.2 Design of conjoint analysis

An adaptive choice‐based conjoint analysis (ACBC) was used in this study. Different from traditional conjoint analysis, the prediction of consumer choices in a choice‐based conjoint analysis is not on product judgment but actual product choices, respondents make a choice or decision, so the need for estimating consumer choices is removed (Jaeger, Hedderley, & MacFie, 2001). The ACBC method has been suggested to be more accurate at measuring consumer response involving five or more attributes and may require fewer respondents than a traditional CBC to obtain similar results (Jervis, Ennis, & Drake, 2012; Orme, 2010).

The ACBC survey was developed in English, translated into the three respective languages, and rechecked by a native speaker. The survey was pretested by five to six respondents for each country after which minor adjustments were made. The online surveys were conducted in the native language of the participants and were held in November to December 2016. The survey began with a short introduction of the research and followed by questions on demographic information and experience in eating fresh and dried mango, for example, frequency of consumption. According to Pollard et al. (2002), experience in consuming a certain food is related to familiarity with that food. Next, the survey comprised three main sections: the first section was designed with one Build‐Your‐Own (BYO) questionnaire in which respondents were introduced to the attributes and levels while they were asked to identify the product closest to their ideal. In the second section, five screening tasks with four product concepts per task with the possible responses of “a must‐have” or “an unacceptable” attribute were created for each product concept. A minimum of two and a maximum of three attributes varied from the BYO selections for each product concept. Two “unacceptable” questions and one “must‐have” question were built into the survey. In the third section–a choice task tournament– they were asked to select the concept that best fitted their preferences from a maximum of 14 product concepts and with a minimum of three concepts per choice task.

Following the conjoint survey, an additional section was added to measure the health consciousness of the respondents. The health consciousness questionnaire (Schifferstein & Ophuis Oude, 1998) assesses whether individuals are aware of the influence of lifestyle on health (Wardle & Steptoe, 2003), and ready to take health actions (Becker, Maiman, Kirscht, Haefner, & Drachman, 1977). Respondents rated 11items of health consciousness on a 5‐point scale ranging from 1 = strongly disagree, 2 = disagree, 3 = neutral, 4 = agree to 5 = strongly agree, see Table S1. Upon completion of the entire survey, respondents were entered into a lottery to receive one of the five €15 voucher cards for each country.

#### 2.1.3 Data collection and respondents

The online survey was created using Sawtooth Software Lighthouse Studio 9.2 (Sawtooth Software Inc., Provo, UT, USA). The survey was circulated by the researchers of this study and their networks to respondents living in the respected countries, such as university students and employees via emails, social media (e.g., Facebook), and personal message applications (e.g., WhatsApp and Line). A total of 638 respondents participated, of which 483 respondents gave valid answers. Answers of respondents who completed the survey in less than 5 minutes, gave repetitive answers (e.g., always choose “agree” for all health consciousness questions, which contained positive and negative statements), and/or did not complete the survey were removed from the database.

### 2.2 Statistical analysis

The data were analyzed by hierarchical Bayesian estimation and rescaled with the zero‐centered difference method using Sawtooth Software Lighthouse Studio 9.2 (Sawtooth Software Inc.). The results–for example, importance scores and utility values within a country, importance scores per attribute between countries–were compared by applying one‐way ANOVA with *post hoc* Hochberg‐GT2 using IBM SPSS Statistics 25 (IBM Corp., Armonk, NY, USA).

The internal consistency reliability of the health consciousness scale was measured with Cronbach's Alpha. The overall reliability was 0.78, which is above the 0.70 level that is generally considered to be satisfactory (Tavakol & Dennick, 2011). The reliability values for each country were also satisfactory: China (0.78), the Netherlands (0.83), and Indonesia (0.71).

## 3 RESULTS AND DISCUSSION

### 3.1 Sociodemographic characteristics

Table 2 shows the sociodemographic characteristics of the three respondent groups: Chinese, Indonesians, and Dutch, and their health consciousness scores. Most of the respondents were females comprising 70.6% of the sample. The age distribution is skewed with 61.7% of the sample population between 18 and 25 years. The respondents were well educated with 78.3% of them holding an undergraduate or postgraduate qualification.

**Table 2:** Average health consciousness scores (from 1=low to 5=high) of respondents according to sociodemographic characteristics and familiarity

	Chinese				Indonesians				Dutch				All respondents			
Characteristics	*n*	Mean	SD	Statistics	*n*	Mean	SD	Statistics	*n*	Mean	SD	Statistics	*n*	Mean	SD	Statistics
	137	3.13	0.54		244	3.47	0.45		102	3.06	0.58		483	3.29	0.54	
*Gender*				*t* = 1.118, *n.s*.				*t* = 1.173, *n.s*.				*t* = 0.962, *n.s*.				*t* = 0.055, *n.s*.
Male	35	3.22	0.47		73	3.54	0.47		34	2.97	0.61		142	3.33	0.56	
Female	102	3.10	0.56		171	3.44	0.43		68	3.11	0.57		341	3.27	0.53	
*Age*				*F* = 0.284, *n.s*.				*F* = 0.778, *n.s*.				*F* = 0.524, *n.s*.				*F* = 4.641, *P < *0.05
18 to 25	64	3.11	0.56		201	3.45	0.44		33	3.12	0.62		298	3.34^a^	0.52	
26 to 40	68	3.16	0.53		38	3.55	0.46		38	2.98	0.56		144	3.22^ab^	0.56	
41 to 60	5	3.00	0.43		5	3.45	0.58		31	3.09	0.57		41	3.13^b^	0.57	
																
*Education level*				*F* = 5.633, *P* < 0.01				*F* = 0.591, *n.s*.	102			*F* = 0.302, *n.s*.	483			*F* = 2.282, *n.s*.
Middle/High school	8	2.53^a^	0.32		58	3.42	0.45		39	3.09	0.61		105	3.23	0.56	
Diploma/Bachelor	69	3.16^b^	0.51		162	3.48	0.45		31	2.99	0.61		262	3.34	0.52	
Master or higher	60	3.18^b^	0.54		24	3.54	0.46		32	3.10	0.55		116	3.23	0.55	
*Experience in eating DM*				*t* = 1.434, *n.s*.				*t* = –1.998, *P* < 0.05				*t* = – 0.246, *n.s*.				*t* = 0.533, *P* < 0.05
Yes (Eater)	126	3.15	0.54		138	3.42^a^	0.43		51	3.05	0.6		315	3.25^a^	0.53	
No (Noneater)	11	2.91	0.47		106	3.53^b^	0.46		51	3.07	0.57		168	3.35^b^	0.55	
*Country*																*F*=28.937, *P* < 0.001
China													137	3.13^a^	0.54	
Indonesia													244	3.47^b^	0.45	
the Netherlands													102	3.06^a^	0.58	

Note: Different letters indicate significant differences between groups following ANOVA post hoc Hochberg‐GT2 at *P* < 0.05.

DM, dried mango.

### 3.2 Health consciousness scores

Indonesian respondents were on average more health conscious than Chinese and Dutch respondents (*P* < 0.05), Table 2. It is worth noting that the average health conscious score of the total respondents (*n* = 483) was rather high, on average 3.3 on a 5‐point scale. This value suggests that the sample population in this study considered themselves as health conscious individuals.

In the total sample, no significant difference in the average health consciousness score was found for gender (Table 2). The youngest respondents appeared to be more health conscious than the oldest respondents (*P* < 0.05). This cannot be attributed to differences within countries since no differences between age groups within the three countries were found, but it seems due to the high number of young Indonesians in the sample. There is no significant difference in health consciousness scores between educational backgrounds within a country, except for the Chinese respondents, where lower educated respondents have lower health consciousness scores. However, some caution is necessary due to the small number of Chinese respondents in the sample with a lower educational background.

A significant correlation exists between the health consciousness score and being a dried mango eater (*P* < 0.05), as shown in Table 2. This result might be because the number of Indonesian respondents contributed to more than half of the total respondents. Indonesian groups who are less health conscious (as compared with those who have higher health conscious scores) tend to be dried mango eaters (*P* < 0.05). This result is in line with our previous health perception study, which found that Indonesian participants ate dried mango as a snack and their reasons for eating were often unrelated to health (Sulistyawati et al., 2019). Nevertheless, it should be noted that the Indonesian groups have the highest health consciousness scores among the three respondent groups (*P* <0.05). For Dutch and Chinese respondents, no significant difference in the health consciousness between dried mango eaters and noneaters was found, implying that for these groups dried mango consumption might not be associated with a healthy diet and lifestyle. The results of this study were different from previous consumer studies on dried fruit (Cinar, 2018; Sijtsema, Jesionkowska, Symoneaux, Konopacka, & Snoek, 2012). Cinar (2018) found a positive relationship between consumers being health conscious and their willingness to purchase dried fruits (banana, kiwi, and pineapple).

### 3.3 Familiarity levels

This study involved respondents from the Netherlands, China, and Indonesia. These countries show large differences in quantity and variety of dried fruit products available on their market, so participants from these countries are expected to have different familiarity with eating dried fruit, specifically dried tropical fruit. Chinese participants were most familiar with dried mango, reflected in 94.9% of the respondents being dried mango eaters, followed by Indonesian (64.3%) and Dutch participants (50%), Table 2. These results are in line with the consumer perception study on dried mango in which Chinese and Indonesian participants mentioned more intrinsic characteristics (Sulistyawati et al., 2019), implying a higher product familiarity than the Dutch participants.

For Indonesians, 35.7% of noneaters of dried mango is remarkably low. This might be due to the fact that various alternatives of fruit snacks are available in the Indonesian market ranging from various fresh tropical fruits (Altendorf, 2017, 2018) to processed fruit products, including semidried/dried fruits.

It is worth noting that although Dutch respondents were least familiar with eating dried mango among the groups studied, the proportion of the eaters is still rather high (50%). This can be explained by the recent increase in semidried/dried mango popularity and higher availability as snack and ingredient in breakfast cereals and patisseries. Nevertheless, half of the Dutch respondents–who do not eat dried mango–might be hampered by the limited availability of dried mango in the Dutch supermarkets and greengrocers. According to the Europe Health Interview Survey conducted between 2013 and 2015, more than 85% of Dutch people regularly consume fresh fruits and vegetables, respectively (EUROSTAT, 2018), which might also contribute to lower dried fruit consumption.

### 3.4 Relative importance of intrinsic quality attributes

**Figure 1:**
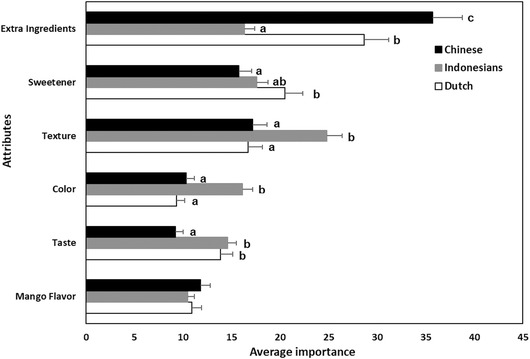
Relative importance of intrinsic attributes (mean ± SE) contributing to consumer preference in dried mango in three countries. Different letters show significant differences per attribute between respondent groups (*P* < 0.05).

This study measured the relative importance of six intrinsic attributes for choosing dried mango and compared the significant differences among the three respondent groups: Chinese, Indonesians, and Dutch (Figure 1). The relative importance scores reflect the importance of each attribute in the decision‐making process for a preferred product concept by the respondents. The attribute *(no) extra ingredients* had the greatest importance in determining consumers’ preference for Chinese and Dutch groups, while for Indonesians, it was the third most important attribute (but not significantly different from *sweetener*), as shown in Figure 1.

*Texture* was the most important attribute for selecting and consuming dried mango for Indonesians, being “crispy” as the preferred level (Table 3 and Figure 1). This finding seems contradictory to the health consciousness scores in which Indonesians had higher scores than other groups, but they weighed texture–a sensory attribute–as the most important attribute in choosing dried mango. For the Chinese and Dutch, this attribute was the second most important intrinsic quality attribute, but not significantly different from *sweetener* (*P* < 0.05). The importance of texture for dried fruit is especially true for dried fruit snacks. A previous texture preference study on dried apple and pear found that the Chinese, Koreans, and the U.S. consumers like crispy samples and dislike soft and jelly‐like samples (Wong, Kim, Chung, & Cho, 2020).

**Table 3:** The most preferred levels per intrinsic attribute of dried mango

Intrinsic attributes	Chinese	Indonesians	Dutch
**Extra ingredients**	**No extra ingredients**	No extra ingredients	**No extra ingredients**
**Sweetener**	Low calorie, natural sweetener		
**Texture**	Chewy	**Crispy**	Chewy
**Color**	Light orange		Yellow
**Taste**	Balanced sweet and sour		More sweet than sour
**Mango flavor**	Similar to fresh mango		

Note: The levels in bold are the key intrinsic quality attributes based on the relative importance of the attributes from the conjoint analysis.

The least important attribute of dried mango was different for the three groups; it was *taste* for the Chinese, *mango flavor* for the Indonesians, and *color* for Dutch respondents (Figure 1). *Taste* usually falls within the top three most important sensory attributes of a food (Prescott, 1998). This result is in line with the relatively high health consciousness of the samples, which can drive a health‐related food choice behavior,that is, making food choice by considering health‐related attributes over the nonhealth‐related attributes (Mai & Hoffmann, 2012). It is worth noting that this study did not include tasting, which can influence the importance rating and utilities (De Pelsmaeker, Schouteten, Lagast, Dewettinck, & Gellynck, 2017).

### 3.5 Utility values for different levels of each attribute of dried mango

**Figure 2:**
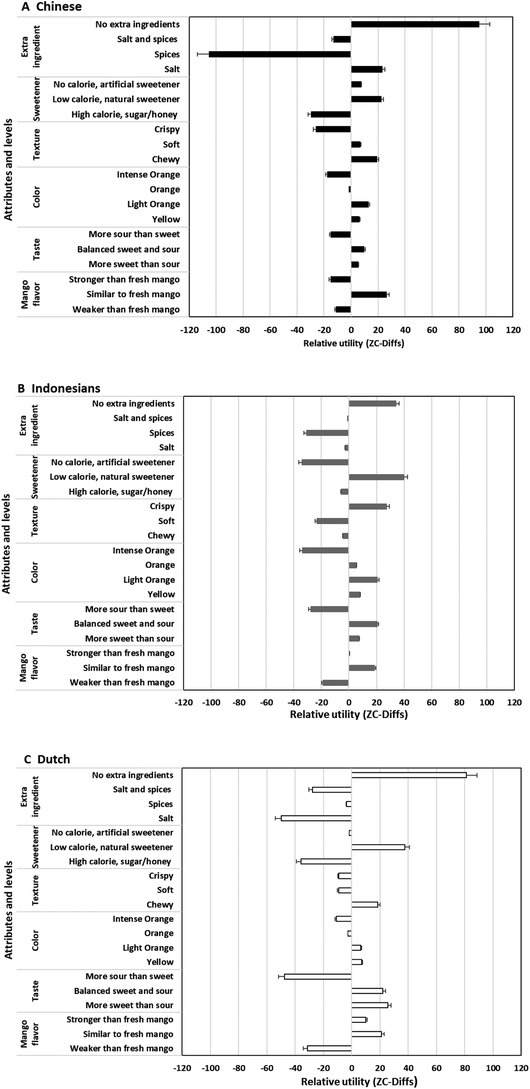
Preference for intrinsic quality attributes and their levels of dried mango in each group of respondents.

Figure 2 shows the utility values for different levels of each intrinsic quality attribute shown in Figure 1. The values reflect the contribution of the respective level to the consumers’ preference for choosing dried mango. “No extra ingredients” was the preferred level for *extra ingredients* in all respondent groups (Figure 2 and Table 3). “No extra ingredients” might be interpreted as a preference for a “natural” product. This finding is in line with a thorough review study on the importance of food naturalness for consumers that revealed that to the majority of consumers, food naturalness is crucial (Román, Sánchez‐Siles, & Siegrist, 2017). Gomez, Schneid, and Delaere (2015) also found that consumers differed in their perception of food naturalness and the difference influenced their consumption of fresh dairy products. This result implies that it can be relevant to take naturalness into account when developing dried mango.

All respondent groups distinctly less preferred adding “salt” or “salt and spices” or “spices.” Adding salt could be seen as turning healthy fruit into unhealthy dried fruit, as it is also recommended to reduce dietary salt intake for the prevention of cardiovascular disease (Hooper, Bartlett, Davey Smith, & Ebrahim, 2004). Moreover, for the Dutch group, as for other European consumers, dried fruit is commonly consumed as “just” dried fruit (e.g., raisin) or incorporated in sweet products like breakfast cereals, muesli bars, and mixed with nuts, as reported by Jesionkowska, Konopacka, Płocharski, Sijtsema, and Zimmermann (2007), implying that adding salt or spices into dried fruit is not a common practice for these consumers.

Despite the rather similar preference among the three groups on “no extra ingredients,” adding salt showed a positive utility value only for the Chinese group. This might be related to the availability of dried salted fruit made of sour plum, mango, and tangerine/orange peel in the local market in China (Liu & Yin, 2015) and high salt intake of the Chinese on a regular basis (Hvistendahl, 2014), implying their preference for salty foods including dried fruit.

“Low calorie, natural sweetener” is the most preferred sweetener for all respondent groups (Figure 2 and Table 3). This is followed by “no calorie, artificial sweetener” for Chinese and Dutch groups, and “high calorie, natural sugar/honey” for the Indonesian group. Following that, the Chinese and Dutch groups significantly preferred “no calorie, artificial sweetener” over “high calorie, natural sugar/honey,” while the Indonesian group preferred the opposite. This could imply that respondents give different importance weights to the level of calorie and type of sweetener, which, however, was not studied here.

A significant difference in texture preferences was found between the groups. The Indonesians appreciated crispy dried mango over a chewy or soft texture, as reflected in the distinct positive utility value (Figure 2 and Table 3). Dutch and Chinese had negative utility values for “crispy,” they preferred “chewy” over the other textures. It is likely that the Indonesian group considers dried mango only as a snack as also reported by Sulistyawati et al. (2019), which is often regarded as indulgence and comfort food (Jack, O'Neill, Piacentini, & Schröder, 1997). Moreover, this finding is in agreement with Oddo, Maehara, and Rah (2019), who revealed that 65% of Indonesian adults consume fried snacks for 4 days/week, suggesting that they repeatedly consume food with a crispy texture. In the study of Sulistyawati et al. (2019), some Indonesian participants reasoned eating dried fruit crisps was for enjoyment and then they preferred adding flavor enhancer. Dutch groups preferred a “chewy” texture perhaps due to their frequent consumption (weekly of more often) of chewy dried fruits, for example, raisin and dried apricot (Jesionkowska et al., 2008). In addition, the Dutch are used to consume dried fruits, including dried mango, as a chewy snack or in breakfast cereals, as can be found in the supermarkets and greengrocers. In China, a wide variety of dried fruit is commonly consumed and is also available as additional ingredients, like in breakfast porridge or baked goods (Wei et al., 2017), thus it could be assumed the dried fruit eaten by the Chinese usually has a “chewy” texture. These findings give an important market insight regarding texture of dried mango.

Regarding *color*, the Chinese and the Indonesians preferred light orange, while the Dutch preferred yellow dried mango (Figure 2 and Table 3). Concerning *taste*, Dutch respondents preferred a “more sweet than sour” taste, while Indonesian and Chinese respondents preferred a “balanced sweet and sour” taste (Figure 2 and Table 3). As expected, a “more sour than sweet” taste gave negative utility values for all respondent groups. Intense yellow orange or orange mango flesh indicates a full ripened mango (Medlicott, N'Diaye, & Sigrist, 1990) and a sweeter taste due to ripening (Yashoda, Prabha, & Tharanathan, 2006). In relation to this, the results on color and taste preferences may imply that all respondents favored dried mango, which resembled the color and taste characteristics of ripe mango. However, it should be noted that the color and taste of dried mango also depend on the mango variety used (Alphonce et al., 2015) and the added ingredients.

No significant difference in the relative importance of attributes was found between men and women (*P* > 0.05). To our surprise, also no significant differences in the relative importance of attributes were found between dried mango eaters and noneaters, which was hypothesized influencing product familiarity. Consumers having a different frequency of consuming certain food are likely to differ in their assessment of that food attributes (Hersleth, Lengard, Verbeke, Guerrero, & Næs, 2011). Results of the present study also differed from a previous consumer study on fresh fruit, which found that high‐frequency fruit consumers (as compared to those of low‐frequency fruit consumers) tended to attach more value to many intrinsic fruit attributes, for example, freshness and nutritional value (Heng & House, 2018).

The results of taste preference of the Chinese and Indonesians are similar to a previous preference study on dried mango, which revealed that Norwegian respondents mostly appreciated a sweet and sour balance of dried mango (Alphonce et al., 2015).

In the present study, preferences of dried mango attributes differ between the three countries studied (Table 3). The key intrinsic attribute in dried mango for the Indonesians is *texture*, while for the Chinese and the Dutch, it is (not adding) *extra ingredients*. Nevertheless, this difference seems not to be influenced by familiarity nor health consciousness. The different preferences for intrinsic attributes of dried mango between groups might be due to factors of cultural differences and individual preferences. As reported by Pollard et al. (2002), these two factors contributed to affecting food choice in relation to fruit and vegetable intake.

### 3.6 Limitations

One limitation of this study is the use of social media and e‐mail to recruit the respondents. As such, the respondents were self‐selected and are not necessarily representative of the general population because they were not divided according to age, gender, or health consciousness. The varied number of respondents per country makes it a challenge to compare them from a statistical point of view.

Another limitation of this study was that we only examined the frequency of dried mango consumption to represent product familiarity. Furthermore, while the health consciousness scale has been tested and applied in several studies (e.g., Chen, 2013; Gámbaro, Ellis, & Prieto, 2013; Sijtsema et al., 2012), this self‐reported health consciousness may be assessed differently by consumers across countries due to variability in health perception. Due to the fact that tasting is–of course–not possible in an online questionnaire, participants may have assessed the preference of sensory attributes of dried mango differently than when they would have had the opportunity to actually taste dried mango. These results must be regarded with caution because a lack of consistency in texture terminology might have existed among participants that potentially resulted in a variation in their interpretation of the levels of the textural attributes.

## 4 CONCLUSIONS AND RECOMMENDATIONS

According to this study, consumer preferences toward intrinsic attributes of dried mango differ between respondent groups from China, Indonesia, and the Netherlands. The key quality attribute considered by the Indonesians is texture, with crispy as the preferred texture, the Dutch and Chinese consider the lack of extra ingredients as the key attribute. They prefer a purer mango product, without “extra ingredients.” This is one of the key intrinsic attributes of dried mango and interpreted to be related to naturalness, suggesting that naturalness needs to be taken into account in product development. The results of this study suggest that adding value to dried mango by addressing different intrinsic quality attributes is relevant. In reality, consumers choose products considering also extrinsic quality attributes, for example, origin, price, and nutritional information (Rodrigues et al., 2017); therefore, further studies are recommended, addressing both types of attributes, intrinsic and extrinsic, for a more comprehensive understanding in choosing dried fruit. The results relate to the preference of intrinsic attributes of dried mango consumed as a single product, not as an ingredient of a food product. Further studies are needed to shed additional light on the understanding preference of dried fruit products, such as protein bar, on‐the‐go dried fruit/nut mixes.

Both familiarity toward dried mango–operationalized as ever/never ate it and local availability–and health consciousness do not influence the preference. To identify factors influencing the consumers’ preference toward dried mango or comparable types of products, a case‐specific approach is necessary for which product familiarity can be investigated involving more indicators (e.g., product knowledge and taste preference).

This study demonstrates the application of conjoint analysis in multiple countries as a valuable tool in product development. The results of this study contribute particularly to explaining variations in key quality attributes and preferences; providing useful insights for food manufacturers to create a more targeted new product development strategy for dried mango and other dried tropical fruits.

## AUTHOR CONTRIBUTIONS

I.S. and B.S. designed the study. I.S. participated in data collection and drafted the manuscripts. I.S., B.S., and M.D. interpreted the results. B.S., M.D., and R.V. supervised the work and reviewed the manuscript.

## CONFLICTS OF INTEREST

All authors report no conflicts of interest.

## Supporting information

Table S1. The health consciousness scale (Schifferstein & Ophuis Oude, 1998) and internal consistency reliability
Fig S1. Relative importance attributes (mean ± SE) contributing to consumer preference of dried mango in each respondent groups.
