# Supplementary material for: Consumer preference for dried mango attributes: A conjoint study among Dutch, Chinese, and Indonesian consumers
Source: J Food Sci. 2020 Sep 12;85(10):3527–35. doi: 10.1111/1750-3841.15439 (PMC7590129; doi:10.1111/1750-3841.15439)
Supplement: Supplementary file 1 — Table S1. The health consciousness scale (Schifferstein & Ophuis Oude, 1998) and internal consistency reliability Fig S1. Relative importance attributes (mean ± SE) contributing to consumer preference of dried mango in each respondent groups. [file JFDS-85-3527-s001.docx]

**Table S1-The health consciousness scale (Schifferstein & Ophuis Oude, 1998) and internal consistency reliability**

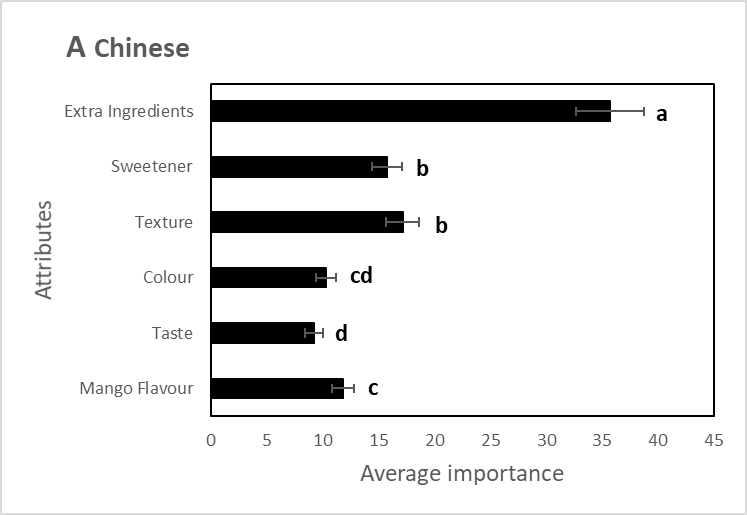


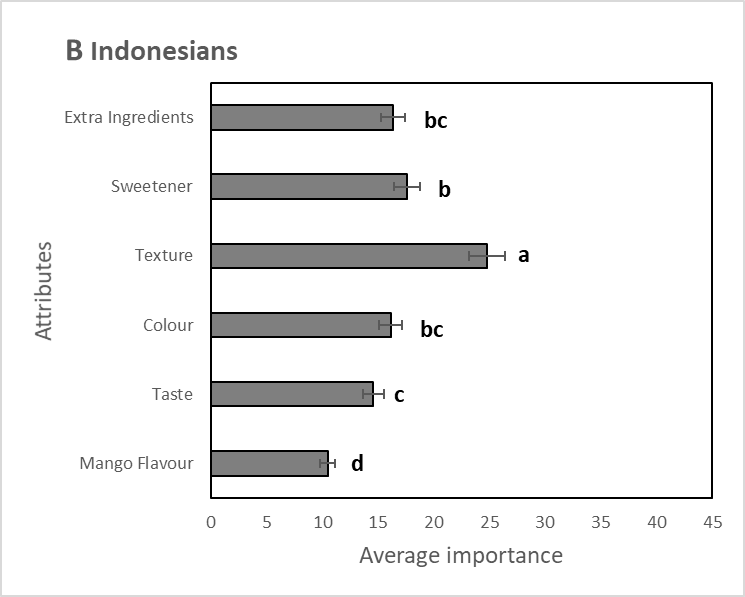


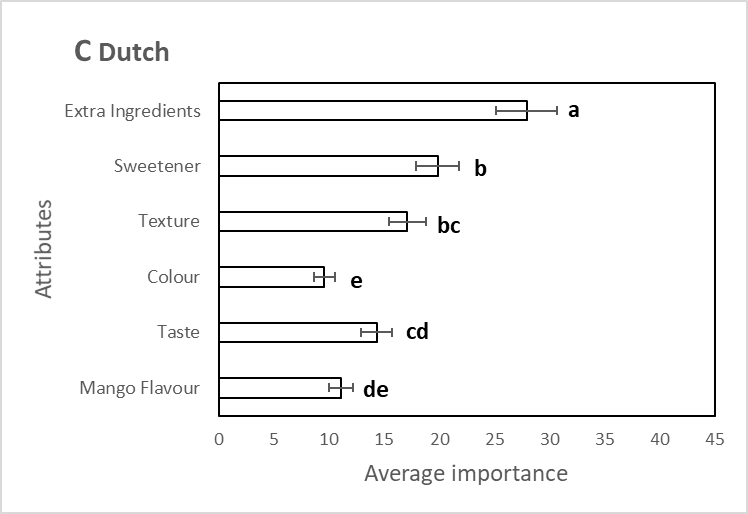


**Fig S1.** Relative importance attributes (mean ± SE) contributing to consumer preference of dried mango in each respondent groups. Different letters show significant differences of an attribute within a group (P < 0.05).
